# A rapid review and synthesis of the effectiveness of programmes initiating community-based antiretroviral therapy in sub-Saharan Africa

**DOI:** 10.4102/sajhivmed.v21i1.1153

**Published:** 2020-11-05

**Authors:** Raymond Chimatira, Andrew Ross

**Affiliations:** 1 Department of Public Health, School of Public Health and Nursing, University of KwaZulu-Natal, Durban, South Africa; 2 Department of Family Medicine, School of Public Health and Nursing, University of KwaZulu-Natal, Durban, South Africa

**Keywords:** community-based ART, HIV, interventions, ART initiation, retention, attrition, viral suppression, sub-Saharan Africa

## Abstract

**Background:**

Community-based antiretroviral therapy initiation (CB-ARTi) has the potential to reduce attrition by increasing access to care, reducing patient costs, decongesting clinics and ensuring improved uptake of ART. There is a paucity of research that identifies successful implementation of CB-ARTi in sub-Saharan Africa (SSA).

**Objectives:**

The aim of the study was to review and describe the evidence on the effectiveness of CB-ARTi programmes that start ART in communities in comparison with the current standards of care in SSA.

**Methods:**

A rapid review of grey and published peer-reviewed literature between January 2009 and July 2019, by using PubMed, PDQ-Evidence, Google Scholar, clinical trial databases and major HIV (human immunodeficiency virus) conference websites, was conducted. Search terms used included ‘community-based’, ‘home initiation community models’, ‘antiretroviral therapy’, ‘clinical outcomes’, ‘viral suppression’, ‘retention in care’, ‘loss to follow-up’, ‘HIV’ and ‘sub-Saharan Africa’.

**Results:**

The search yielded 90 articles and reports following the removal of duplicates. After initial screening and full-text screening, six articles remained and were included in the qualitative narrative synthesis. This included four randomised control trials and two cohort studies of specific interventions comparing CB-ARTi with the standard of care in SSA. There is evidence that CB-ARTi can increase access to HIV-testing services, linkage to ART, retention in care and viral suppression rates and is possibly not inferior to facility-based healthcare.

**Conclusion:**

CB-ARTi has the potential to increase access to HIV services to people living with HIV in SSA. The results mentioned previously suggest that CB-ARTi models could prove to be equal and possibly not inferior to facility-based ones and warrant further investigation.

## Introduction

Sustainable human immunodeficiency virus (HIV) epidemic control requires that a large percentage of people living with HIV (PLHIV) must initiate antiretroviral therapy (ART) early, regardless of their CD4 T-cell count or clinical stage, and remain in care, adhere to treatment and achieve viral suppression.^1^ To this end, the Joint United Nations Programme on HIV and AIDS (UNAIDS) set the ambitious 95–95–95 treatment targets that by 2030, 95% of all PLHIV will know their HIV status, 95% of all those diagnosed as HIV-positive will receive sustained ART and 95% of all those receiving ART will achieve viral suppression.^1^

However, the achievement of the 95-95-95 targets in sub-Saharan Africa (SSA) is challenged by a weak HIV care cascade, with PLHIV being lost at each step as a result of barriers to getting tested, linkage to and staying in care, and starting or adhering to ART.^2^ Many studies from SSA report that rates of linkage to care and initiation of ART in individuals who tested HIV-positive are lower than 50%.^2,3^

Successful strategies to address the high rates of patient attrition at every stage of the HIV care cascade include rapid ART initiation and differentiated care models with community ART distribution for stable patients on ART. Community-based HIV programmes that include dispensing ART have contributed significantly to decongesting the traditional healthcare services, and improved adherence and retention in care.^2,3,4^ Other interventions identified through systematic reviews and meta-analyses include community-based HIV testing, facilitated referrals for ART initiation, education, treatment supporters and active adherence reminder devices such as mobile phone text messages. However, these have shown mixed results, with each intervention also being found not to produce significant effects in some settings.^5^ Despite this, interest is increasing in the role of out-of-facility or community-based ART initiation (CB-ARTi) as an essential approach for universal access to HIV care.

Community-based ART initiation has the potential to reduce attrition by increasing access to care, reducing patient costs, decongesting clinics and ensuring improved uptake of ART.^3,6,7^ There is a need for the updating of the status of evidence that supports the implementation of CB-ARTi models. Such evidence should include its impact on the clinical and behavioural outcomes such as retention in care and viral suppression amongst patients initiating ART in SSA. The objective of this article is to review and describe the evidence of the effectiveness of CB-ARTi programmes that start ART in communities in comparison with the current standard of care in SSA, namely the initiation of ART in traditional facility-based hospitals and clinics.

## Methodology

This rapid review used a streamlined systematic method to capture the evidence from current community-based approaches to the initiation of ART in SSA. There were two research questions. (1) What are the essential elements of evidence-based models of CB-ARTi that inform policy in SSA? (2) How do the reported clinical outcomes, for example, retention in care and viral suppression, amongst patients initiating ART in community-based settings compare with traditional standards of care in SSA?

The rapid review approach streamlines traditional systematic review methods to gain efficiency and accelerate the review process, whilst still aiming to produce valid conclusions.^8^ We focussed on a narrow topic, used a limited rather than an exhaustive range of search terms and restricted the analysis and synthesis. We also restricted the search of grey literature (material written for professionals and disseminated outside of peer-reviewed journals) to key websites and only considered studies published in English since January 2009. Furthermore, we performed single (vs. dual) screening of titles and abstracts by using Rayyan, a web-based semi-automated screening software (https://rayyan.qcri.org/welcome).

## Search strategy

Studies were identified through bibliographic searches of PubMed and PDQ-Evidence publication databases by using the following terms and variations: ‘community based’; ‘home initiation’; ‘community models’; ‘home care services’; ‘health facility’; ‘clinic’; ‘antiretroviral therapy’; ‘antiretroviral’; ‘clinical outcomes’; ‘patient adherence’; ‘patient compliance’; ‘viral load’; ‘viral suppression’; ‘retention’; ‘retention in care’; ‘loss to follow-up’; ‘attrition’; and ‘HIV’. The search strategy was limited to studies conducted in SSA and published in English from January 2009 through July 2019. The PubMed search strategy is summarised in Box 1.

BOX 1 PubMed search string.

#1 ‘community-based’ OR ‘home initiation’ OR ‘home-based’ OR ‘community models’ OR ‘home models’ OR ‘home care services’ OR ‘facility-based’ OR ‘health facility’ OR ‘hospital’ OR ‘clinic’ OR ‘referral and consultation*’
#2 ‘retention in care’ OR ‘retention’ OR ‘loss to follow up’ OR ‘patient compliance’ OR ‘patient adherence’ OR ‘viral suppression’ OR ‘viral load’ OR ‘clinical outcomes’ OR ‘Kaplan-Meier estimate’ OR ‘attrition’
#3 HIV OR ‘hiv seropositivity/diagnosis’ OR ‘hiv infections/diagnosis’ OR ‘hiv infections/virology’ OR ‘AIDS’
#4 ‘antiretroviral’ OR ‘antiretroviral therapy’ OR ‘antiretrovir*’ OR ‘ART’ OR ‘anti-HIV agents’ OR ‘therapeutics’ OR ‘initiation’
#5 ‘sub-Saharan Africa’ OR ‘Africa south of the Sahara’

HIV, human immunodeficiency virus; AIDS, acquired immune deficiency syndrome; ART, antiretroviral therapy.

We also conducted a grey literature search that was limited to abstracts from the following major HIV-related conferences: Conference on Retroviruses and Opportunistic Infections (CROI), the International AIDS Conference, International Conference on AIDS and STDs in Africa (ICASA), Southern African HIV Clinicians’ Society Conference and the South African AIDS Conference. In addition, we searched clinical trial databases for planned or ongoing research via the U.S. National Library of Medicine Register of Clinical Trials (clinicaltrials.gov) and the WHO (World Health Organisation) International Clinical Trials Registry Platform (ICTRP). Three technical experts on HIV care and treatment were also contacted by e-mail for comment on the appropriateness of the identified literature and additional articles.

## Study inclusion criteria

Studies were included in the review if they: (1) reported on community-based models of ART initiation; (2) measured clinical outcomes (e.g. linkage to care, adherence, reported stigma, retention in care and virologic suppression) of patients initiated on ART in the community compared with patients receiving the current standard of care; and (3) used observational and experimental methods with primary data within randomised controlled trials (RCTs), cross-sectional or cohort (prospective and retrospective) designs. We excluded articles that did not meet all three criteria from the review.

## Study selection and data extraction

All references captured by the search engine were uploaded to Rayyan and duplicates were identified and removed. The eligibility of the articles was assessed in two steps, the first entailing all titles and abstracts being assessed by the primary reviewer. In the second step, full texts for all the articles deemed relevant to the research questions were read in full and analysed to confirm their eligibility. The primary reviewer extracted data from all eligible articles by using a standard data collection form to collect information about the publication date, study setting, design and interventions, patient population and outcome measures. The secondary reviewer reviewed all the extractions for accuracy.

## Quality evidence assessment and risk of bias

The quality of evidence and risk of bias (RoB) of the studies included in the final review was assessed by one reviewer by using the McMaster University’s Quality Assessment Tool (https://merst.ca/ephpp-tools/) from the Effective Public Health Practice Project (EPHPP). The secondary reviewer verified the quality of evidence and the RoB assessment.

### Ethical consideration

This article followed all ethical standards for research without direct contact with human or animal subjects.

## Results

### Study selection and characteristics of included studies

**FIGURE 1:**
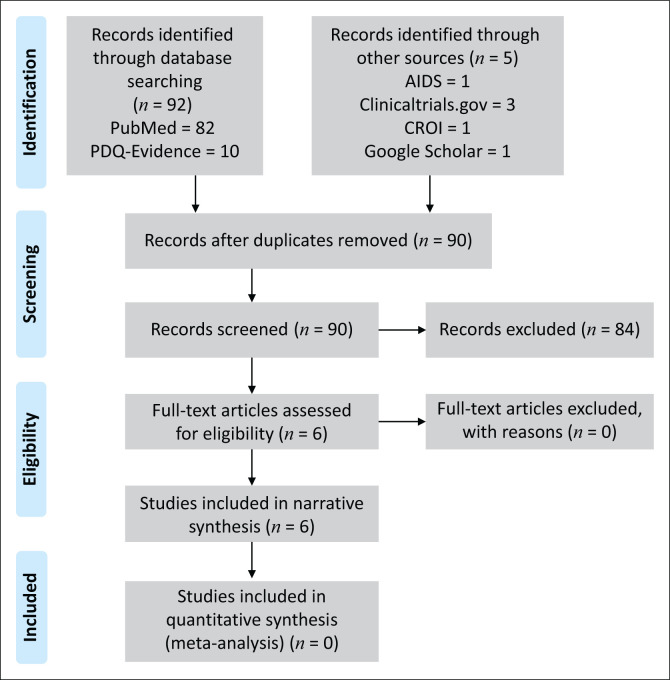
Preferred Reporting Items for Systematic Reviews and Meta-Analyses (PRISMA) flow for study selection.

The process of study identification and selection concluded with 90 citations being identified after removing duplicates (Figure 1). After screening the titles and abstracts, six full-text articles were selected for critical review (Table 1). These consisted of four completed studies and two ongoing RCTs (Table 2). Four studies were included for the qualitative synthesis of patient outcomes, which varied between studies, and included case finding (HIV seropositive yield), linkage to ART initiation, ART adherence, retention in care and viral suppression (Table 3).

**TABLE 1:** Description of studies included in the review.

Author and publication year	Study design	Country and setting	Participants (eligibility criteria)	Sample size (*n*)	Participants and/or healthcare service description		Outcomes measured
					Intervention	Comparator	
MacPherson et al. 2014^4^	Cluster RCT	Malawi urban slums	Adult males and females; ≥ 16 years	244	HIV self-testing (HIVST), optional home initiation of HIV care (including 2 weeks of ART if eligible). Follow-up appointment at their nearest HIV care clinic	HIVST, facility referral for initiation of HIV care (including ART if eligible)	Cumulative incidence of ART initiation; uptake of HIVST; rates of loss from ART at 6 months
Barnabas et al. 2016^6,7^	RCT	South Africa Uganda	Adult males and females; ≥ 18 years	1200 (still recruiting in South Africa)	(1) Home ART initiation and mobile van ART monitoring and resupply (2) Hybrid model with on-site community ART initiation and facility referral ART monitoring and resupply	Clinic ART initiation, monitoring and resupply – the current standard of care (SOC)	Proportion of HIV-positive persons who initiate ART and achieve viral suppression; cost per HIV-positive person with suppressed HIV viral load (VL) at 12 months
Labhardt et al. 2018^3,12^	RCT	Lesotho rural	Adult males and females; ≥ 18 years *Exclusion criteria*: history of previous combination ART exposure, pregnancy, breastfeeding, WHO clinical stage 4, chronic condition (i.e. tuberculosis or diabetes), positive cryptococcal antigen test	278	Same-day home-based ART initiation (*n* = 138) and subsequent follow-up intervals of 1.5, 3, 6, 9 and 12 months after treatment initiation at the health facility	SOC (*n* = 140) with referral to the nearest health facility for preparatory counselling followed by ART initiation and monthly follow-up visits thereafter	Rates of linkage to care within 3 months (presenting at the health facility within 90 days after the home visit); viral suppression at 12 months (VL < 100 copies/mL from 11 through 14 months after enrolment)
Oladele et al. 2018^11^	Retrospective secular trend study; non-randomly assigned local government areas into clusters providing intervention and standard of care (time series)	Nigeria	Intervention: 14 local government areas (districts); control: 34 local government areas (districts)		Model A (on-site initiation) clusters: offered services within communities, from HIV diagnosis to immediate ART initiation and some follow-up.Model B (immediate referral) clusters: offered services for HIV diagnosis up to baseline evaluation and provided referral for ART initiation to nearest health facility	Selected and cluster-matched 34 local government areas where community antiretroviral treatment delivery was not implemented	Number of people identified as HIV positive; number of HIV-positive individuals started on antiretroviral treatment
Tun et al. 2019^9^	Quasi-experimental prospective cohort study	Tanzania rural	Adult females ≥ 18 years who sold sex for money or goods in the past 6 months *Exclusion criteria:* WHO clinical stages 3 or 4 (with symptoms)	509	Comprehensive SRH services for female sex workers (FSWs), including community-based HIV-testing services (HTS), ART initiation and ART delivery	Comprehensive SRH services for FSWs, including community-based HTS, referral to local facilities for ART initiation and ART delivery	Linkage to care (ART initiation) at 6 months; retention in care at 6 months
Amstutz et al. 2019^10^	Cluster-randomised clinical trial	Lesotho rural	Children and adults > 10 years	Estimated enrolment: 262	Same-day home-based ART initiation, village-based ART visit and/or refill, individually customised SMS	Same-day home-based ART initiation, clinic-based ART visit and/or refill, no SMS	Viral suppression; linkage to care; retention in care; all-cause mortality

RCT, randomised controlled trial; ART, antiretroviral therapy; SRH, sexual and reproductive health; SMS, short message service; WHO, World Health Organisation.

**TABLE 2:** Comparative summary of community antiretroviral therapy initiation models identified.

Step in HIV care cascade	Home initiation and immediate referral to healthcare facilities for ART monitoring and resupply in Malawi (MacPherson et al.^4^)	Same-day home initiation and immediate referral to healthcare facilities for ART monitoring and resupply in Lesotho (Labhardt et al.^3,12^)	On-site ART initiation, some community-based ART monitoring and referral to facilities for ART monitoring and resupply in Nigeria (Oladele et al.^11^)	On-site ART initiation and community-based ART monitoring and resupply for FSWs in Tanzania (Tun et al.^11^)	On-site ART initiation and community-based ART monitoring and resupply in Uganda and South Africa – DO ART study (Barnabas^6,7,13^)	Same-day home initiation and community-based ART monitoring and resupply and clinic-based laboratory evaluation in Lesotho – VIBRA study (Amstutz et al.^10^)
**Demand creation**	Counsellors promoted HIVST and home initiation during door-to-door visits.	Mobile outreach teams offered HTS to all household members and offered same-day ART initiation to clients who tested positive.	Community mobilisation and community outreach services.	Brochures and announcements at targeted healthcare facilities and peer support groups.	Following community sensitisation, participants will be recruited through community-based HIV testing and counselling (HTC) and HIV clinics.	Campaign teams visit rural villages to offer HTS and multidisease screening and prevention.
**Patient access for HTC**	Clients self-presented to collect HIVST kits and report test results. If positive, study nurses conducted home visits for ART preparation and home initiation.	Mobile outreach teams conducted door-to-door testing and service provision. If positive, study nurses conducted home visits for ART preparation and home initiation.	Mobile outreach teams conducted door-to-door testing and service provision including immediate ART initiation and some follow-up.	Mobile outreach teams conducted community-based HTC in FSW hotspots; as well as return to care campaigns for FSWs living with HIV but not yet on ART.	Lay counsellors conduct community-based HTC at home or through a mobile van.	Lay counsellors provide home-based HTC.
**Providers**	Counsellors marketed HIVST and home initiation and linked positive clients for home initiation by study nurses. Nurses dispensed 2 weeks of ART if client was eligible.	Counsellors provided home-based HIV testing and linked positive clients for same-day home initiation by study nurses. Nurses dispensed 1 month supply of ART if client was eligible.	Mobile outreach teams included clinicians (medical officers, nurses), lay counsellors, pharmacists and/or other cadre licensed to dispense ART.	Mobile outreach teams included clinicians, nurses and peer educators.	Mobile outreach teams include nurses and lay counsellors.	Mobile teams consist of nurses and lay counsellors.
**Laboratory and clinical evaluation**	Clinical evaluation and CD4 cell count (venous blood for laboratory testing) conducted at first home visit.	Clinical evaluation by study nurse; laboratory evaluation including CD4 cell counts, creatinine levels and haemoglobin by using point-of-care (POC) equipment.	Clinical evaluation by mobile team physician; laboratory evaluation by using POC equipment or sample referral. Same day results.		Clinical evaluation done by using a clinical questionnaire; laboratory evaluation by using POC testing for CD4 count, pregnancy and creatinine.	Clinical evaluation (medical history and physical examination) done by using a checklist; laboratory evaluation by using POC testing for CD4 count, haemoglobin and creatinine.
**Adherence preparation**	Adherence preparation at first and second home visits, if client was eligible for ART.	Clients received pre-ART counselling directly after testing, plus a leaflet that summarised the key points of ART adherence.	Clients completed three sessions at identification in the community. Case managers assigned for immediate follow-up starting same day.	Standard three sessions of adherence counselling at community-based HIV testing and counselling (CBHTC) sites.		Study nurse conducts a one-on-one structured education and counselling session by using a leaflet (5–10 min).
**Place and time of ART initiation**	Within the client’s home, at the second home visit if client was eligible.	Within the client’s home, at the first visit (same day).	At point of identification within the community. ART initiated immediately after completing three sessions of adherence preparation. Could take up to 3 h.	At CBHTC sites, after completing three sessions of adherence preparation.	At the point of identification within community. ART commenced immediately based on clinical and eligibility assessment.	Within the client’s home, at the first visit (same day).
**Follow-up care (and enrolment)**	Clients provided with completed ART registration and follow-up appointment at their nearest HIV care clinic.	Clients instructed to visit their health facility within 2–4 weeks for their follow-up.	Phone calls or SMS or home visits every 3 days during the first 2 weeks. Follow-up visits to community team at 2 weeks and 1 month after ART initiation. Linked to fixed facility for subsequent follow-up after 1 month.	Drug pick-ups at client’s convenience at CBHTC sites. Peer educators ensured regular contact with clients through text messaging and WhatsApp, as well as through monthly meetings to assess adherence and adverse events, and provide peer support.	Participants pick up their medication refills from the mobile van and have all clinical monitoring conducted in the mobile van.	Follow-up visits at health facility *(control arm)* or with the village health worker (VHW) *(intervention arm)* at 12–16 days based on patient preference. VHWs check adherence and provide monthly refills (intervention arm), with patients attending the clinic at 6 and 12 months for laboratory assessment. Individually tailored SMS for adherence and based on VL results.

HIV, human immunodeficiency virus; FSW, female sex worker; HIVST, HIV self-testing; HTS, HIV testing services; ART, antiretroviral therapy; SMS, short message service; VL, viral load; VIBRA, village-based refill of ART.

**TABLE 3:** Effectiveness of community antiretroviral therapy initiation models identified.

Step in HIV care cascade	Home initiation and immediate referral to healthcare facilities for ART monitoring and resupply in Malawi (MacPherson et al.^4^)	Same-day home initiation and immediate referral to healthcare facilities for ART monitoring and resupply in Lesotho (Labhardt et al.^3,12^)	On-site ART initiation, some community-based ART monitoring, and referral to facilities for ART monitoring and resupply in Nigeria (Oladele et al.^11^)	On-site ART initiation and community-based ART monitoring and resupply for FSWs in Tanzania (Tun et al.^9^)
**HIV test uptake and case finding**	No significant difference in the uptake of HIVST kits between home and facility groups. Participants in home group were more likely to report a positive HIVST result (6.0%) than the facility group (3.3%). Median CD4 cell count at ART initiation was highest amongst home initiators (219c/uL) compared with facility initiators (154c/uL).	No data reported	Both Model A (on-site initiation) and Model B (immediate referral) clusters had more HIV positive identified per 100 000 population in the 12 months after community-ART introduction compared with the 12 months before (Model A: 11 374 vs. 5352; and Model B: 907 vs. 152)	No data reported
**Linkage to care and ART initiation**	The cumulative incidence of ART initiation was significantly higher in the home group (2.2% of residents) compared with the facility group (0.7% of residents).	Linkage to care within 90 days after enrolment was higher in the same-day group (68.6%; 94/137) compared with the usual care group (43.1%; 59/137).	Both Model A (on-site initiation) and Model B (immediate referral) clusters had more HIV positives initiated on ART per 100 000 population in the 12 months after commART introduction compared with the 12 months before (Model A: 7347 vs. 2181; and Model B: 499 vs. 152). For Model A cluster, 59.6% of HIV positives identified in health facilities were linked to ART compared with 69.1% of HIV positives identified in the community.For Model B cluster, 80.9% of HIV positives identified in health facilities were linked to ART compared with 31.6% of HIV positives identified in the community.	At 6 months, 256/256 (100%) of the intervention group and 181/253 (71.5%) of the comparison group were linked to care and on ART.
**Retention in care**	At 6 months, 52/181 (28.7%) of the home group and 15/63 (23.8%) of the facility group were lost to follow-up. In unadjusted analysis, the rate of loss to follow-up was higher amongst the home group (63.4/1000 person-months) than in the facility group (53.5/1000 person-months).	At 12 months, 12/137 (8.8%) of same-day group and 10/137 (7.3%) of usual care group were lost to follow-up.	No data reported.	At 6 months, 254/254 (100%) of the intervention group and 171/180 (95%) of the comparison group remained in care and on ART.
**Viral suppression**	No data reported.	At 12 months (11–14 months), 69/137 (50.4%) of same-day group and 47/137 (34.3%) of the usual care group achieved documented viral suppression (VL < 100 copies/mL). In each group, 14/137 (10.2%) had no documented VL, the remaining not attending health facilities within that time frame.	No data reported.	No data reported.
**Other**	None	At 6 months, 51/137 (37.2%) of same-day group and 36/137 (26.3%) of usual care group achieved documented viral suppression (VL < 100 copies/mL).	None	Less likely to report high levels of internalised stigma.

HIV, human immunodeficiency virus; ART, antiretroviral therapy; FSW, female sex workers; HIVST, HIV self-testing; VL, viral load.

We excluded all studies that did not report on ART initiation in the community (out-of-facility). Interventions were implemented in Lesotho, Malawi, Nigeria, South Africa, Tanzania and Uganda. The four completed studies were assessed by using the McMaster University’s Quality Assessment Tool, and individual studies ranged in quality from 1 (strong) to 3 (weak), with the overall average being 2. The two ongoing RCTs included in the model description were not assessed for quality as they had not been completed at the time of reporting.

### Models of community-based antiretroviral therapy initiation

Community-based ART initiation modalities include home, mobile and workplace as part of an HIV-testing campaign. The review identified two main models of CB-ARTi, with some variations across countries (Table 2): (1) on-site ART initiation and community-based ART monitoring and resupply^7,9,10^ and (2) a hybrid model with on-site community ART initiation and referral to local clinics for ART monitoring and resupply.^3,4,11^ In addition, the review identified key activities addressing seven areas of CB-ARTi service delivery, namely (1) demand creation, (2) patient access for HTC, (3) provider roles, including task shifting, (4) laboratory and clinical evaluation, (5) adherence preparation, (6) place and time of ART initiation and (7) follow-up care.^3,4,9^ Examples of these CB-ARTi programme activities, including summaries of information on the purpose of the activity, populations targeted and strategies used, are provided in Table 2.

### Effects on clinical outcomes

Two completed RCTs,^3,4^ one quasi-experimental prospective cohort study^9^ and one retrospective interrupted time series cohort study^11^ were included for the qualitative synthesis of patient outcomes. Two of the studies reported on HIV test uptake and case finding, all four on linkage to ART initiation, three on retention in care and one on viral suppression (Table 3).

#### Human immunodeficiency virus test uptake and case finding

One RCT reported on HIV test uptake, whilst one RCT and one retrospective interrupted time series cohort study reported on HIV case finding as an outcome. In urban slums in Malawi, MacPherson et al.^4^ reported that there was no significant difference in the uptake of HIV-self-testing (HIVST) kits between offering optional home initiation of HIV care after self-testing (home group) compared with HIVST followed by facility-based services only (facility group). However, participants in the home group were more likely to report a positive HIVST result (6.0%) compared with the facility group (3.3%). In addition, the median CD4 T-cell count at ART initiation was higher amongst home (219 cells/µL) than facility initiators (154 cells/µL).

Oladele et al.^14^ found that introducing two models of community ART delivery services resulted in more HIV-positive individuals being identified per 100 000 population in 14 high-burden local government areas in Nigeria in the 12 months after the models were introduced compared with the 12 months before. Model A (immediate on-site initiation) identified 11 374 versus 5352 per 100 000 population, whilst Model B (HIV diagnosis up to baseline evaluation and referral for ART) identified 907 versus 152 per 100 000 population. Furthermore, preliminary data from the delivery optimization for antiretroviral therapy (DO ART) study^13^ suggest that 80% (320/398) of persons testing HIV positive in rural Uganda were eligible for same-day ART initiation, with men accounting for more than half the persons eligible (169/320; 53%).

#### Linkage to antiretroviral therapy initiation

Four studies reported on linkage to ART initiation as an outcome. The study by MacPherson et al.^4^ in urban slums in Malawi found that the cumulative incidence of ART initiation was significantly higher in the home (2.2% of residents) compared with the facility group (0.7% of residents). Labhart et al.^3^ found that linkage to ART within 90 days after enrolment was higher in the same-day home-based ART initiation group (94/137; 68.6%) than in the facility-based care group (59/137; 43.1%) in rural Lesotho. Tun et al.^11^ found that at 6 months, 256/256 (100%) of the community-based intervention group and 181/253 (71.5%) of the facility-based comparison group self-reported as being linked to care and on ART amongst sex workers in Tanzania.

Oladele et al.^14^ found that both Model A (on-site initiation) and Model B (immediate referral) clusters had more HIV positives initiated on ART per 100 000 population in the 12 months after the models were introduced compared with the 12 months before (Model A: 7347 vs. 2181; and Model B: 499 vs. 152). For Model A clusters, 59.6% of HIV positives identified in health facilities were linked to ART compared with 69.1% of HIV positives identified in the community. For Model B clusters, 80.9% of HIV positives identified in health facilities were linked to ART compared with 31.6% of HIV positives identified in the community.

#### Retention in care and loss to follow-up

Three studies reported on retention in care or loss to follow-up as an outcome. MacPherson et al.^4^ found that at 6 months, 52/181 (28.7%) of the home group and 15/63 (23.8%) of the facility group were lost to follow-up. In addition, the rate of loss to follow-up was higher amongst the home group (63.4/1000 person-months) than in the facility group (53.5/1000 person-months) in unadjusted analysis. Labhardt et al.^3^ found that at 12 months, 12/137 (8.8%) of the same-day community-based group and 10/137 (7.3%) of the facility-based care group were lost to follow-up. Retention in care since enrolment was significantly higher in the same-day community-based group (*p* = 0.009). Tun et al.^11^ found that at 6 months, 254/254 (100%) of the intervention group and 171/180 (95%) of the comparison group remained in care and on ART.

#### Viral suppression

One RCT reported on viral suppression as an outcome. Labhardt et al.^3^ reported that ART-naïve participants who were assigned to the same-day home-based ART initiation were more likely to remain in care at 12 months and achieve VL suppression. The authors reported that in the 11 to 14-month window after enrolment, 69/137 (50.4%) of the same-day group and 47/137 (34.3%) of the facility-based care group achieved documented viral suppression (VL < 100 copies/mL). In each group, 14/137 (10.2%) had no documented VL, whilst the remaining patients did not attend the health facility within that time frame.

### Effects on behaviour

Two studies measured self-reported medication adherence, whilst one measured self-reported internalised stigma and an ongoing one reported on patient acceptability.

#### Medication adherence

MacPherson et al.^4^ found that, based on clients completing an adherence questionnaire (at 2–4 weeks, 3 months and 6 months), 19/164 (11.6%) and 3/60 (5.0%) ART initiators in the home and facility groups, respectively, self-reported missing at least one dose of ART in the past 4 days at any assessment point (*p* = 0.14). Tun et al.^9^ found that medication adherence was not significantly different amongst those with a completed adherence questionnaire, with 37/214 (17.3%) and 25/152 (16.4%) self-reported missing at least one dose of ART in the past 7 days, and 2/214 (0.9%) of the same-day group and 9/159 (5.7%) of the usual care group self-reported stopping taking ART for more than 30 days continuously (*p* = 0.008).

#### Internalised stigma and acceptability

Tun et al.^9^ used a validated six-item scale to assess participants’ feelings of shame and guilt as a result of living with HIV and found that a community-based intervention group was less likely to report high levels of internalised stigma compared with the facility-based group (26.6% vs. 39.9%; *p* = 0.001). This supports the findings by Wyatt et al.^6^ who reported that amongst 50 DO ART study participants in Uganda, home initiation was associated with decreased concerns about disclosure risk at facilities. The authors also reported that other participants perceived home initiation and community follow-up to have many advantages compared with facility-based care, including being convenient, saving time and money otherwise spent on travel to clinics, and being responsive to individual needs. Additional benefits reported include reaching hard-to-reach populations, for example, FSWs,^9^ men at trading posts and those only available in the evenings and at weekends.^3,6,13^

### Cost analysis

MacPherson et al.^4^ reported that the average cost of the home-based ART services was US$97.11 per patient assessed. In comparison, the average cost per patient initiated on ART was US$172.46. Data on estimated cost per participant from the DO ART study in Uganda and South Africa are still pending.^7^

## Discussion

### Summary of the evidence

A rapid review method was used to quickly capture the current evidence on the essential elements of evidence-based models of community-based (out-of-facility) ART initiation and the reported outcomes amongst patients initiating ART in community-based settings in SSA. We searched two databases, five conference websites and two registers of clinical trials for intervention studies evaluating CB-ARTi models, with four completed and two ongoing studies being included in this review.

The review identified heterogeneity in interventions, study design, location and definition of outcomes measured as a major obstacle to interpreting and synthesising the data on CB-ARTi. For example, some of the authors report on retention in care and loss to follow-up at 6 months^4,9^ versus 12 months,^3^ and linkage to care at 3 months^3^ versus 6 months.^9^ These differences are highlighted in Tables 1 and 2.

However, despite the limitations noted above, the existing data suggest that CB-ARTi could be more effective in increasing the uptake of HIV testing and improving case finding at a population level^4,11^ than in facility-based ART initiation. Other advantages include improved linkage to ART initiation,^3,4,9,11^ which results in similar rates of viral suppression.^3^ One study conducted before the universal test and treat (UTT) era reported a higher rate of loss to follow-up amongst the home group,^4^ and two other studies conducted in the UTT era reported significantly higher retention in care rates in the same-day group.^3,9^ In addition, two studies found that there was no difference in self-reported medication adherence,^3,9^ and one reported low self-reported internalised stigma.^9^ The above results suggest that CB-ARTi models are equal and certainly not inferior to facility-based healthcare.

### Strengths and benefits of community-based antiretroviral therapy initiation

Community-based HTS are an essential pillar towards reaching HIV epidemic control. However, it is estimated that almost two-thirds of patients are lost in the process from community-based HIV testing to facility ART initiation without specific interventions, with higher loss to follow up (LTFU) rates amongst African cohorts.^14^ Hence, many authors and technical experts believe that CB-ARTi has considerable potential to address the gap between HIV diagnosis and ART initiation.^3,9,10,11^ The strengths of the CB-ARTi models noted in the literature include reducing the structural barriers, such as cost of transport to the clinic, time saved otherwise spent on travel to clinics, flexibility of hours and location of service delivery and addressing stigma associated with traditional healthcare (especially for key populations), which in turn appear to result in better access to ART initiation for ‘hard-to-reach’ populations, such as men, FSWs and other key populations.^3,6,11,14^

## Concerns and barriers related to community-based antiretroviral therapy initiation

There are concerns that CB-ARTi may shift scarce resources and ART initiations from healthcare facilities, which may result in their being unsustainable.^4,9^ However, CB-ARTi identified patients with higher median CD4 cell counts, which in turn may influence cost-effectiveness favourably through reduced morbidity and mortality.^4^ In addition, studies in Malawi and Nigeria both reported that the rates of facility-based ART initiations remained stable, whilst community-based initiations provided extra numbers.^4,11^ Another issue raised in the literature relates to the potential for increased rates of loss to follow-up,^4,11^ which means that additional adherence support measures should be put in place as CB-ARTi moves from small pilot studies to programmatic scale-up.

Concerns have also been expressed about the perceived lack of confidentiality, especially in smaller communities.^4^ However, participants from Uganda and Tanzania reported CB-ARTi to be an acceptable option that is perceived to have many advantages compared with facility-based care.^6,9,13^

## Knowledge gaps and future directions

The purpose of this rapid review was to synthesise and describe what is currently known on the topic of CB-ARTi. Based on the findings, several considerations for future research and practice in the field of CB-ARTi are evident. Firstly, it is essential to consider the fit of CB-ARTi initiatives within the context of the local epidemic conditions to ensure that they complement existing healthcare systems. This includes addressing existing facility-level barriers, such as long waiting times and poor staff attitudes, to increasing uptake of ART.

Secondly, given the results, which suggest that CB-ARTi models are possibly not inferior to facility-based ones, it is crucial to revisit existing policies on decanting stable virally suppressed patients to community ART distribution models after 12 months. This is especially important for key and other priority populations, such as men, adolescents and young people whose retention in care is often hindered by facility-level barriers.

Thirdly, it is important to consider the needs of the population by developing partnerships with community leaders and community-based organisations to overcome potential barriers related to lack of confidentiality, and widespread stigma and discrimination. In addition, designing CB-ARTi initiatives that are integrated into general health campaigns can also address the potential lack of confidentiality. Community-based ART initiation models should therefore include assessments for non-communicable diseases, screening for sexually transmitted infections and tuberculosis (TB), HIV pre-exposure prophylaxis (PrEP) and other HIV prevention services, family planning services, as well as alcohol and drug rehabilitation services. Finally, further research is needed regarding the impact and estimated costs, as well as the cost-effectiveness of CB-ARTi models.

### Limitations

Our results and conclusions might be susceptible to the bias related to the limits of a rapid review. These include restricting the search to literature published in English language and two electronic databases (although we used the PubMed database that contains by far the most significant number of health and medical journals). The search was also complemented by grey literature searches from major HIV-related conferences, clinical trial databases and brief technical expert consultations.

A second limitation is that only one author screened and selected the titles and abstracts from the total set of documents retrieved, extracted data from all eligible articles and assessed the quality of evidence and RoB. However, this author is knowledgeable about the content area. The second author checked all the extractions for accuracy and verified the quality of evidence and RoB assessment.

As a result of the heterogeneity in intervention, study design, location and definition of outcomes measured, we were not able to combine the results to estimate overall intervention effect or draw conclusions on the relative effectiveness of community-based versus facility-based ART initiation. Varying definitions of the outcomes of interest also impacted the comparability of the results. This major obstacle has been highlighted by Rosen et al.^15^ who have proposed standardised primary and secondary outcomes for research on accelerating ART initiation. Finally, some of the studies assessed are still incomplete, and the final results with higher numbers of participants (higher statistical power) may differ significantly from some of the preliminary findings presented.

## Conclusion

This rapid review identified a small but rich set of information on the topic of CB-ARTi. However, after weighing the existing evidence, it appears that there is evidence that CB-ARTi can increase access to HTS, linkage to ART, retention in care and viral suppression rates, and is possibly not inferior to facility-based healthcare. The results reached earlier suggest that CB-ARTi models could prove to be equal and possibly not inferior to facility-based ones and warrant further investigation. The apparent promise and pitfalls of CB-ARTi and the increasing interest of policymakers in its potential as a strategy to increase linkage to care and ART uptake in the era of UTT indicate that careful monitoring of the evidence base is warranted.
